# Chronotype: what role in the context of gastroenteropancreatic neuroendocrine tumors?

**DOI:** 10.1186/s12967-021-03010-1

**Published:** 2021-07-30

**Authors:** Luigi Barrea, Giovanna Muscogiuri, Gabriella Pugliese, Roberta Modica, Daniela Laudisio, Sara Aprano, Antongiulio Faggiano, Annamaria Colao, Silvia Savastano

**Affiliations:** 1 Dipartimento di Scienze Umanistiche, Università Telematica Pegaso, Via Porzio, Centro Direzionale, isola F2, 80143 Naples, Italy; 2 Centro Italiano per la cura e il Benessere del paziente con Obesità (C.I.B.O), Department of Clinical Medicine and Surgery, Endocrinology Unit, University Medical School of Naples, Via Sergio Pansini 5, 80131 Naples, Italy; 3 Dipartimento di Medicina Clinica e Chirurgia, Unit of Endocrinology, Federico II University Medical School of Naples, Via Sergio Pansini 5, 80131 Naples, Italy; 4 Endocrinology Unit, Department of Clinical and Molecular Medicine, Sant’Andrea Hospital, Sapienza University of Rome, 00189 Rome, Italy; 5 Cattedra Unesco “Educazione alla salute e allo sviluppo sostenibile”, University Federico II, Naples, Italy

**Keywords:** Chronotype, Gastroenteropancreatic tumors (GEP-NET), Metabolic syndrome (MetS), Tumor aggressiveness, Diet, Nutrition, Nutritionist

## Abstract

**Background:**

Chronotype is defined as a trait determining the subject circadian preference in behavioral and biological rhythms relative to external light–dark cycle. Although individual differences in chronotype have been associated with an increased risk of developing some types of cancer, no studies have been carried out in gastroenteropancreatic neuroendocrine tumors (GEP-NET).

**Materials:**

We investigate the differences in chronotype between 109 GEP-NET and 109 healthy subjects, gender-, age-, and BMI-matched; and its correlation with tumor aggressiveness.

**Results:**

GEP-NET patients have a lower chronotype score (*p* = 0.035) and a higher percentage of evening chronotype (*p* = 0.003) than controls. GEP-NET patients with morning chronotype had lower BMI, waist circumference, and higher percentage of MetS (*p* < 0.001) than evening type. Interestingly, considering the clinical pathological characteristics, patients with the presence of metastasis, grading G2, and in progressive disease presented the lower chronotype score (*p* = 0.004, *p* < 0.001, and *p* = 0.002; respectively) compared to other categories. Chronotype score was negatively associated with anthropometric measurements, metabolic profile, percentage of MetS, and Ki67 index (*p* < 0.001 for all).

**Conclusions:**

GEP-NET patients have an unhealthy metabolic profile and present more commonly an evening chronotype. These results support the importance of including the assessment of chronotype in an adjunctive tool for the prevention of metabolic alterations and tumor aggressiveness of GEP-NET.

## Background

Neuroendocrine tumors (NETs) are a heterogeneous group of tumors with different clinical manifestations and biological behavior. These tumors originate from neuroendocrine cells dispersed throughout the entire body in the endocrine system. Among NETs there are the neuroendocrine neoplasms of unknown primary origin [1], and the tumors of the appendix, usually detected incidentally after appendectomy [2]. The first approach recommended in patients with localized neuroendocrine neoplasms is the surgery [3]. However, most NETs patients already have advanced disease at the time of clinical presentation and therefore require medical therapy [4–7]. The gastroenteropancreatic (GEP) neuroendocrine tumors (NETs) represent instead the most common subtype of NETs [8].

The prevalence and incidence of gastroenteropancreatic neuroendocrine tumors (GEP-NET) is increasing over the past 2 decades, partly due to the growing availability of advanced endoscopic and radiological imaging techniques in detecting more accurately benign and incidentally discovered lesions [9]. The complex clinical presentation and course of GEP-NET deserves special attention in early diagnosis and treatment, particularly in their association with metabolic consequences that represent risk determinants of well-differentiated GEP-NET, similarly to other types of cancers [10]. Thus, the management of patients with GEP-NET requires an integrated multidisciplinary approach, and nutrition is an essential component of the assessment and management of these patients [11].

In the frameshift of the worldwide increase in chronic, degenerative and inflammatory diseases, it is well-established the crucial role of nutrition in cancer onset, progression, and outcome, and the consumption of healthy foods and nutrients are recognized as a valid strategy for primary cancer prevention, in particular in gastrointestinal neoplasms [12–15]. Besides the traditional approach in controlling quantity and the quality of dietary intake, there is an increasing interest in the chrononutrition, the relationship between dietary intake and the circadian clock, in cancer patients. Foods and timing of food intake are among the synchronizing factors able to lead to circadian desynchronization, with a negative impact on their health; in 2019 WHO’s International Agency for Research on Cancer determined that shiftwork involving circadian disruption is a probable human carcinogen [16].

The core clock machinery is a biochemical system synchronized with the cycles of light and dark and in cells is composed of an autoregulatory network consisting of positive and negative transcription-translation feedback loops [17]. In particular, transcription factors CLOCK/BMAL1 regulate expression of key circadian genes including Cryptochromes (CRY1 and CRY2) and Period (PER1, PER2, and PER3) genes, which are the negative regulators of the circadian loop. While Molecular understanding of Clock, BMAL1, CRY, and PER family functions in circadian regulation are sufficiently known, data are emergent with regard to the molecular basis of tumor-suppressive functions [18]. About Cell-cycle cross-talk, studies have demonstrated that circadian clock components can induce or repress cell-cycle progression depending on the time of day, inducing rhythmic transcriptional and posttranscriptional control of the cell cycle. Recent evidence also established direct relationship between the core circadian clock and apoptosis [19]. Similar to what was observed with cell-cycle regulation, circadian factors can both promote and restrict apoptosis, dependent on cellular context and clock status [20]. With respect to promoting cell death, for example, CRY1/2 and PER1 influence the extrinsic TNFα-dependent pathway and intrinsic apoptotic pathways, respectively [20].

Chronotype is the attitude of a subject in determining individual circadian preferences in behavioral and biological rhythms relative to the circadian rhythm [21]. There are three general categories of chronotypes: morning, evening, and intermediate chronotypes. Morning chronotype uses to wake up early and tend to carry out activities earlier in the day; conversely, the evening chronotype usually wakes up later and prefers to time peak activity during the late afternoon or evening. Intermediate chronotype has intermediate characteristics between the morning and evening chronotype [22]. Delay in meal timing and a habit of skipping breakfast has been often detected in subjects with the evening chronotype [23]. Evening chronotype has been reported to have more health problems such as psychological disorders, gastrointestinal diseases, and greater mortality compared to the morning chronotype [24]. In addition, evening chronotype has been identified as a risk factor for cancer. In a case–control study nested within the California Teachers Study (CTS) cohort, 39.686 postmenopausal participants were enrolled and provided information on the chronotype by completing a questionnaire in 2012–2013 [25]. The 2.719 cases developed primary invasive breast cancer diagnosed from 1995/1996 through completion of the chronotype questionnaire, while 36.967 CTS participants (controls) remained cancer-free during this same time period. Compared to morning type, evening type had an increased risk of breast cancer [25]. In the same CTS cohort were also evaluated the incident cases of endometrial cancer (437 endometrial cancer cases and 26.753 cancer-free controls) and it was also confirmed for this tumor that women who were defined evening types compared to morning types, had a statistically significantly elevated OR of endometrial cancer (95% CI 1.09–1.91) [26]. Similar findings were reached by Papantoniu et al., that investigated the association of chronotypes with the risk of prostate cancer [27]. One thousand and ninety-five prostate cancer cases and 1.388 randomly selected population controls were enrolled and were assessed by face-to-face interviews and chronotype through a validated questionnaire. Risk of prostate cancer was higher among subjects with an evening chronotype, but interestingly also increased in the morning chronotype after long-term night work [27]. A case control case study (496 cases with epithelial ovarian cancer and 906 controls) on shift work exposure, a factor influencing the circadian clock, in relation to epithelial ovarian cancer risk was also conducted and it was observed that more than half of the cases (53.4%) and controls (51.7%) worked evening and/or night shifts, moreover, there was no clear pattern of increasing epithelial ovarian cancer risk with increasing years of shift work [28].

Although there is evidence that evening chronotype is associated with an increased risk of developing cancer, there are no studies that investigate the association of chronotype with the severity of cancer type.

Based on this background, this cross-sectional, case–control observational study aims to investigate the differences in chronotype in GEP-NET patients than a control group matched for gender, age, and BMI. In addition, we investigated also the role of chronotype categories within the context of GEP-NET, in particular, on the clinical severity of disease.

## Materials and methods

### Design and setting

In this cross-sectional case–control observational study, both GEP-NET patients and controls were enlisted from May 2017 to January 2020 at European Neuroendocrine Tumor Society (ENETS) Center of Excellence Multidisciplinary Group for Neuroendocrine Tumors, Department of Clinical Medicine and Surgery, Unit of Endocrinology, University “Federico II” of Naples. The study was approved by our Ethical Committee (n. 201/17) and was conducted following the Code of Ethics of the World Medical Association (Declaration of Helsinki) for human studies. All patients were informed about the study design and purpose and signed informed consent.

### Population study

A total of 218 adult Caucasian subjects were enrolled in this study, of whom 109 GEP-NET patients and 109 healthy volunteers of the same geographical area as the control group among hospital volunteers and in the OPERA (Obesity, Programmes of nutrition, Education, Research and Assessment of the best treatment) prevention project [29]. The control group were matched by gender, age, and BMI. In the choice of the control group, subjects with concomitant liver or renal insufficiency, alcohol abuse, chronic inflammatory diseases, history of cancer, or on pharmacological therapy were excluded; as well as individuals who simultaneously participated in other studies, to avoid influencing the results. The enrolled patients had these characteristics in order to make the study more accurate and homogeneous:

- Confirmed histological diagnosis of well-differentiated, low grade G1 and G2 GEP-NET, according to the classification of by the WHO [30], that recognized three forms of well differentiated NET based on the proliferative activity expressed by the Ki67 index: NET G1 (< 3%), NET G2 (3–20), NET G3 (> 20%) and a form of poorly differentiated NET the neuroendocrine carcinoma G3;
- Functioning GEP-NET: biochemically free of disease, without pharmacological treatment, or after endoscopic surgery carried out more than 6 months before enrollment;
- Non-functioning GEP-NET: at the moment of diagnosis treatment-naïve or after endoscopic surgery carried out more than 6 months before the enrollment, or discontinuing somatostatin analogues for more than 6 months;
- Instead, the exclusion criteria for GEP-NET patients are shown below:
- Confirmed histological diagnosis of well-differentiated/high grade G3 GEP-NET or poorly differentiated neuroendocrine carcinomas, according to WHO classification [30], since these more aggressive cancers have been reported to be associated with malnutrition and cachexia [31];
- Confirmed histological diagnosis of Merkel cell carcinoma, pheochromocytoma/paraganglioma, medullary thyroid cancer, bronchial or thymic NET;
- Any pharmacological treatment, even including somatostatin analogues or targeted therapy, as they can alter the sense of hunger, or induce anorexia, and intestinal motor, secretory and absorptive function as well as liver function [32];
- History of major abdominal surgery that may alter intestinal anatomy;
- Patients with functioning GEP-NET who have not undergone gastrointestinal curative surgery for less than 6 months before recruitment; and that were not pharmacologically treated at the moment of recruitment with drugs that affect the secretion of hormones (peptides and amines) could cause dysfunction of the gastrointestinal tract, including altered motility, diarrhea, steatorrhea, and malabsorption [32];
- Presence of serious diseases or that can alter the metabolism and intestinal function such as concomitant liver or renal insufficiency, alcohol abuse, chronic inflammatory diseases, and history of cancer.

### Physical activity and smoking habits

A standard questionnaire, already reported in other studies [33, 34], was used to evaluate physical activity, reporting if the subjects performed habitually at least 30 min/day of aerobic exercise (YES/NO). Participants were also classified into ‘non-current smokers’, ‘current smokers’ when smoking at least one cigarette per day and ‘former smokers’ when stopped smoking at least 1 year before enrollment, as previously reported [35, 36]. In the analysis, both former and noncurrent smokers were considered as no-smokers.

### Anthropometric measurements

Height and body weight were obtained on participants wearing light clothes and without shoes using, respectively, a wall-mounted stadiometer to the nearest 1 cm and a calibrated balance beam scale derived to the nearest 50 g (Seca 711; Seca, Hamburg, Germany). BMI was calculated by weight and height using the formula: weight (kg) divided by height squared (m^2^), kg/m^2^. According to BMI, subjects were classified as normal weight (BMI 18.5–24.9 kg/m^2^), overweight (BMI 25.0–29.9 kg/m^2^), grade I obesity (BMI 30.0–34.9 kg/m^2^), grade II obesity (BMI 35.0–39.9 kg/m^2^), grade III obesity (BMI ≥ 40.0 kg/m^2^), as previously reported and in accordance with the WHO’s criteria [37–41]. According to the National Center for Health Statistics (NCHS), WC was obtained using a non-stretchable measuring tape to the closest 0.1 cm at the narrowest point or, if no narrowest point was visible, at a midway level between the lower edge of the rib cage and the iliac using a non-stretchable measuring tape [42], as already reported [43, 44].

### Blood pressure and criteria to define MetS

The systolic (SBP) and diastolic (DBP) blood pressure were measured three times in all subjects after 10 min of rest in a sitting position using a random sphygmomanometer (Gelman Hawksley Ltd., Sussex, UK), as explained in other previous studies [45, 46], and the mean of the second and third measurements were reported. According to the National Cholesterol Education Program Adult Treatment Panel (NCEP ATP) III definition [47], if three or more of the following criteria were present [WC ≥ 102 cm (men) or 88 cm (women), blood pressure ≥ 130/85 mmHg, triglycerides level ≥ 150 mg/dL, high-density lipoprotein (HDL) cholesterol level ≤ 40 mg/dL (men) or ≤ 50 mg/dL (women), and glycemia levels ≥ 100 mg/dL], the diagnosis of MetS was confirmed.

### Assay methods

All samples were collected between eight and 10 a.m. after an overnight fast of at least 8 h, and preserved at − 80 °C until being analyzed. All biochemical analyses were carried out in the Central Biochemistry Laboratory of our Department, with a Roche Modular Analytics System. Low-density Lipoprotein (LDL) cholesterol and HDL cholesterol were processed by a direct method (homogeneous enzymatic assay for the direct quantitative determination of LDL and HDL cholesterol).

### Assessment of chronotype

The Chronotype assessment of the participants was obtained with the Morningness-Eveningness questionnaire MEQ [22], consisting of 19 multiple-choice questions, each having four or five response options about sleep habits and daily performance. For example, some items concern the time of the day when one feels most productive due to physical or intellectual activities, the time of the day when one is most tired, and the time when one would prefer to wake up to have maximum energy. From the sum of the individual items a score is obtained that ranges from 16 to 86. Based on their scores, individuals were categorized as morning (59–86), neither (42–58), or evening (16–41) chronotype. The self-reported duration of sleep was obtained from the answer to the question “How many hours did you usually sleep per day during the last month?”.

### Clinical and pathological characteristics of the tumor

Clinical and pathological characteristics were obtained for all patients, including tumor stage at diagnosis, primary tumor site and size, mitotic rate, Ki67 index, metastases, familiar history of multiple endocrine neoplasia (MEN)-1, hormonal secretion, comorbidity, treatment and follow-up, were collected for all patients. Tumor stage at diagnosis was categorized in accordance with the ENETS criteria [48], classified in subjects with localized disease (stage I-III) or with advanced disease (presence of metastases, stage IV).

Primary tumor site and size was measured in mm and defined as the maximum tumor diameter in the pathological sample if the patient has undergone surgery, or in the last imaging test [computed tomography scan / magnetic resonance imaging] if the subject did not undergo surgery. In the case of MEN-1 with multiple pancreatic nodules, the diameter of the biggest one was considered for the tumor size. Only in three cases, the primary lesion was not found and the tumor size was not obtained. The mitotic rate and Ki67 index was obtained according to ENETS criteria [48], as previously reported [49]. Formalin-fixed paraffin-embedded tissue samples derived from biopsy or surgery of the primary tumor and/or metastases were used for immunohistochemistry analysis for chromogranin A, synaptophysin and Ki67 index [50]. After classifying the type of GEP-NET according to WHO classification [30], only patients with well-differentiated/low grade GEP-NET, graded as G1 (Ki67 index ≤ 2% and mitoses < 2) or G2 (Ki67 index 3–20% and mitoses 2–20) were enrolled in this study [30].

Participants were classified as ‘disease free’, when after surgical removal there was no biochemical or morphological presence of the disease, and with ‘stable disease’ or ‘progressive disease’ in accordance with RECIST 1.1 criteria [51].

### Statistical analysis

The data distribution was evaluated by Kolmogorov–Smirnov test and the abnormal data were normalized by logarithm. The chi-square (χ^2^) test was used to determine the statistically significant differences in the frequency distribution, including gender, cigarette smoking habit, physical activity, BMI categories and presence of MetS between GEP-NET patients and controls.

The differences in continuous variables between the two groups were compared using the Student's paired *t*-test (chronotype score, presence/absence metastasis, and grading G1/G2) while the differences in multiple groups (morning type, neither type, and evening type; disease status) were analyzed by ANOVA test followed by the Bonferroni post-hoc test. The correlations among age, anthropometric measurement, blood pressure, metabolic profile, MetS (number parameter), and Ki67 index with chronotype score were performed using Pearson *r* correlation coefficients. A partial correlation was performed to control for effect of BMI as confounding factor on the chronotype score. Proportional odds ratio (OR) models, *p*-value, 95% interval confidence (IC), and R^2^, were performed to assess the association among grading G1 *vs* G2, and presence/absence of metastasis. A multinomial logistic regression analysis, χ^2^, p-value, and akaike information criterion (AIC), and R^2^ was performed to model the association among three groups of disease status (disease free, stable disease and progressive disease) with chronotype score. In addition, receiver operator characteristic (ROC) curve analysis was performed to determine area under the curve (AUC), criterion, sensitivity and specificity, standard error, and 95% IC as well as cut-off values for chronotype score in detecting presence of metastasis, high tumor grading (G2), and progressive disease in the GEP-NET patients. Values ≤ 5% were considered statistically significant. Variables with a variance inflation factor > 10 were excluded to avoid multicollinearity. Data were analyzed using the SPSS Software (PASW Version 21.0, SPSS Inc., Chicago, IL, USA) and MedCalc® package (Version 12.3.0 1993-2012 MedCalc Software bvba-MedCalc Software, Mariakerke, Belgium).

## Results

The study population consisted of 109 GEP-NET patients (F:M = 56:53), aged 57.1 ± 16.0 years and 109 healthy individuals matched for gender, age, and BMI. Ninety-seven patients (89.0%) had nonfunctioning GEP-NET, 32 patients (20.2%) had a MEN-1 syndrome. The mean of Ki67 index was 3.88 ± 4.08%. According to the Ki67 index, GEP-NET patients had been classified in grade 1 and 2 (G1 and G2). In particular, 65 patients (59.6%) were grading G1 and 44 patients (40.4%) were grading G2. According to the presence/absence of metastasis, 27 patients (24.8%) had metastases. In particular, metastases had the following localization: 11 patients (10.1%) pancreatic, eight patients (7.3%) intestinal, two patients (1.8%) gastric, and six patients (5.5%) had metastasis with unknown site. Finally, based on the RECIST1.1 criteria, 51 patients (46.8%) were stable disease, 21 patients (19.3%) were in progressive disease, and 37 patients (33.9%) were disease free.

In Table 1 were shown the differences in study parameters between patients with GEP-NET and the control group. As reported, the habit of cigarette smoking was lower in the control group in comparison with GEP-NET patients (*p* < 0.001). Although there was no difference in BMI, the rate of overweight was higher in the control group (*p* = 0.028). Of interest, WC was higher in both males and females with GEP-NET than in the control group (*p* = 0.049 and *p* = 0.008; respectively). In addition, GEP-NET patients presented a higher SBP (*p* = 0.007), a worse metabolic profile, and higher presence of MetS (*p* = 0.001).

**Table 1:** Differences in study parameters between patients with GEP-NET and control group

Parameters	GEP-NET patients n. 109	Control group n. 109	*p-value
Demographic characteristics			
Gender (Males)	53 (48.6%)	53 (48.6%)	χ^2^ = 0.02, *p* = 0.892
Age (years)	57.1 ± 16.0	56.2 ± 12.9	0.370
Clinical characteristics			
Smoking (No)	72 (66.1%)	42 (38.5%)	χ^2^ = 16.54, ***p* < 0.001**
Physical activity (No)	60 (55.0%)	55 (50.5%)	χ^2^ = 0.29, *p* = 0.587
Anthropometric measurement			
BMI (kg/m^2^)	27.6 ± 5.3	28.2 ± 4.1	0.364
Normal weight (n, %)	38 (34.9%)	27 (24.8%)	χ^2^ = 2.19, *p* = 0.099
Overweight (n, %)	36 (33.0%)	53 (48.6%)	χ^2^ = 4.86, ***p*** = **0.028**
Grade I obesity (n, %)	29 (26.6%)	22 (20.2%)	χ^2^ = 0.92, *p* = 0.337
Grade II obesity (n, %)	5 (4.6%)	6 (5.5%)	χ^2^ = 0.01, *p* = 1.000
Grade III obesity (n, %)	1 (0.9%)	1 (0.9%)	χ^2^ = 0.51, *p* = 0.478
WC Males (cm)	97.4 ± 13.5	92.9 ± 10.3	**0.049**
WC Females (cm)	90.5 ± 15.2	84.1 ± 9.8	**0.008**
Blood pressure			
SBP (mmHg)	125.2 ± 12.0	120.5 ± 12.4	**0.007**
DBP (mmHg)	76.7 ± 7.7	75.5 ± 7.9	0.209
Metabolic profile			
Glycemia levels (mg/dL)	108.1 ± 15.5	92.4 ± 14.6	< **0.001**
Total cholesterol (mg/dL)	190.9 ± 41.8	159.0 ± 30.9	< **0.001**
Triglycerides (mg/dL)	127.1 ± 51.6	109.7 ± 28.8	**0.003**
LDL cholesterol (mg/dL)	118.7 ± 40.0	86.7 ± 31.2	< **0.001**
HDL cholesterol (mg/dL)	46.8 ± 15.3	50.3 ± 8.1	**0.034**
MetS			
Number parameter	2.1 ± 1.5	1.0 ± 1.1	< **0.001**
Mets presence	40 (36.7%)	15 (13.8%)	χ^2^ = 14.00, ***p*** **= 0.001**

GEP-NET: Gastroenteropancreatic tumors; BMI: body mass index; WC: waist circumference; SBP: systolic blood pressure; DBP: diastolic blood pressure; HDL: high-density lipoprotein; LDL: low-density lipoprotein; MetS: metabolic syndrome

*A *p*-value in bold type denotes a significant difference (*p* < 0.05)

**Fig. 1:**
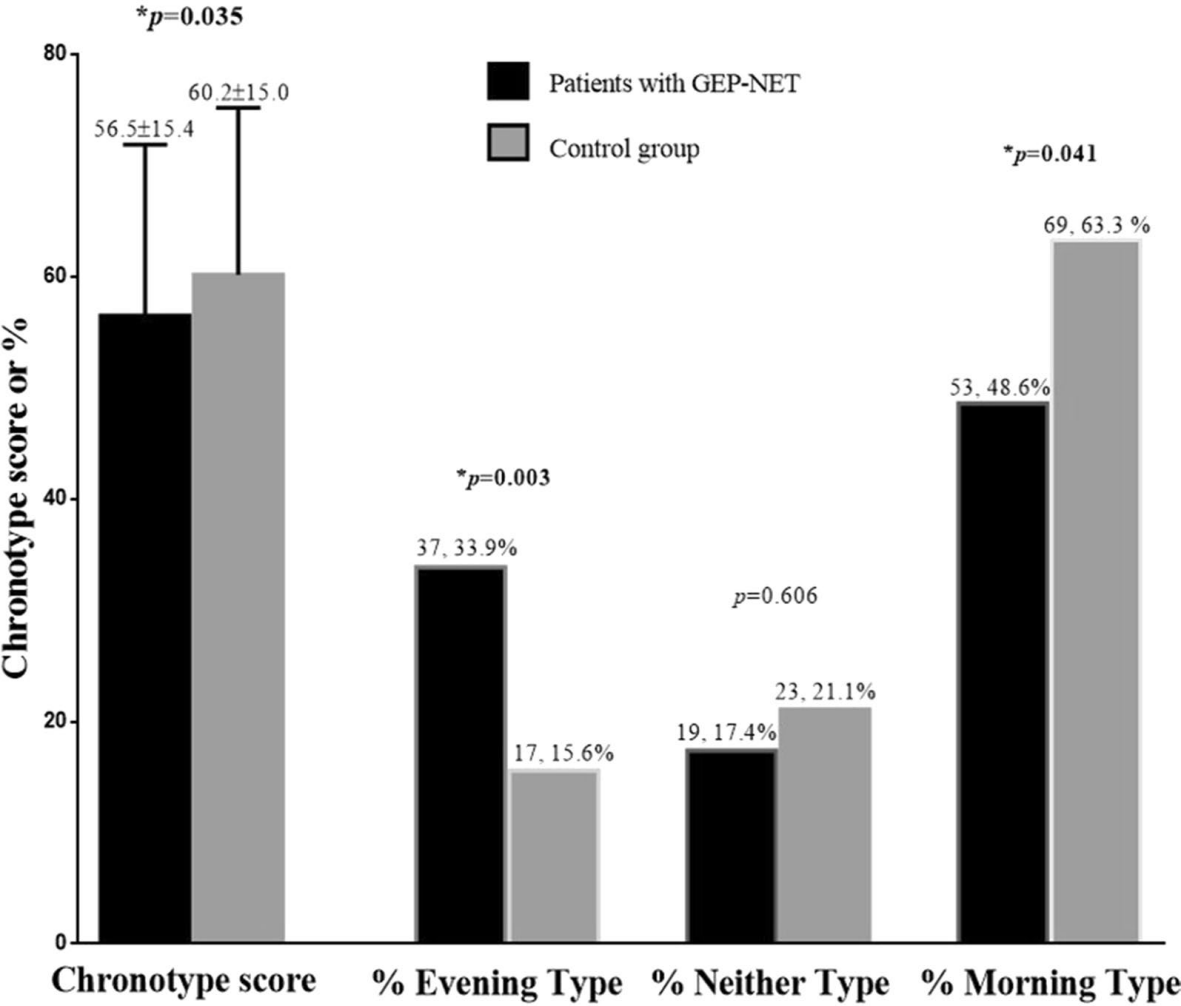
The individual differences in chronotype score and percentage of chronotype in GEP-NET patients compared to the control group. GEP-NET patients have a lower chronotype score and a higher percentage of evening chronotype than controls. In addition, grouped GEP-NET patients according to chronotype categories, most of the participants had a morning chronotype (n = 53, 48.6%), followed by evening type (n = 37, 33.9%), and finally the neither chronotype (n = 19, 17.4%). Difference in chronotype score was analysed by paired Student’s *t* test, while differences in chronotype categories were analysed by chi-square (χ^2^) test. *A *p* value in bold type denotes a significant difference (*p* < 0.05). GEP-NET, gastroenteropancreatic-neuroendocrine tumors

Figure 1 reports the individual differences in chronotype score and percentage of chronotype in GEP-NET patients compared to the control group. Of note, GEP-NET patients have a lower chronotype score (*p* = 0.035) and a higher percentage of evening chronotype (*p* = 0.003) than controls. Grouped GEP-NET patients according to chronotype categories, most of the participants had a morning chronotype (n = 53, 48.6%), followed by evening type (n = 37, 33.9%), and finally neither chronotype (n = 19, 17.4%).

Table 2 summarized the differences in study parameters in GEP-NET patients, according to chronotype categories. A lower percentage of GEP-NET patients with morning chronotype had smoking habits (*p* = 0.023) and was sedentary (*p* = 0.002) compared to intermediate and evening types. In addition, GEP-NET patients with morning chronotype had lower BMI, WC, SBP, DBP, glycemia levels, total cholesterol, triglycerides, and LDL cholesterol, and higher HDL cholesterol and percentage of MetS (*p* < 0.001 for all variables) than evening type.

**Table 2:** Differences in study parameters of GEP-NET, according to chronotype categories

Parameters	Morning type n = 53, 48.6%	Neither type n = 19, 17.4%	Evening type n = 37, 33.9%	*p-value
Gender				
Males (n, %)	25, 47.2%	10, 52,6%	18, 48.6%	χ^2^ = 0.17, *p* = 0.919
Females (n, %)	28, 52.8%	9, 47.4%	19, 51.4%	
Age (years)	54.0 ± 16.6	62.4 ± 12.2	58.7 ± 16.1	0.110
Clinical characteristics				
Smoking (No)	40, 75.5%	14, 73.7%	18, 48.6%	χ^2^ = 7.59, ***p*** =** 0.023**
Physical activity (No)	24, 45.3%	7, 36.8%	29, 78.4%	χ^2^ = 12.73, ***p*** = **0.002**
Anthropometric measurement				
BMI (kg/m^2^)	25.4 ± 3.9	27.6 ± 4.5	30.5 ± 6.1	**< 0.001**
Normal weight (n, %)	26, 49.1%	5, 26.3%	7, 18.9%	χ^2^ = 9.46, ***p* = 0.009**
Overweight (n, %)	18, 34.0%	8, 42.1%	10, 27.0%	χ^2^ = 1.33, *p* = 0.514
Grade I obesity (n, %)	9, 17.0	5, 26.3%	15, 40.5%	χ^2^ = 6.19, ***p*** =** 0.045**
Grade II obesity (n, %)	0,0%	1, 5.3%	4, 10.8%	χ^2^ = 5.84, ***p*** =** 0.050**
Grade III obesity (n, %)	0,0%	0, 0%	1, 2.7%	χ^2^ = 1.96, *p* = 0.375
WC (cm)	87.6 ± 13.2	94.0 ± 13.3	103.0 ± 12.9	< **0.001**
Blood pressure				
SBP (mmHg)	119.2 ± 8.9	124.7 ± 13.8	133.9 ± 9.4	< **0.001**
DBP (mmHg)	73.1 ± 6.7	77.1 ± 7.9	81.8 ± 6.1	< **0.001**
Metabolic profile				
Glycemia levels (mg/dL)	103.5 ± 16.0	101.4 ± 14.3	118.2 ± 9.3	< **0.001**
Total cholesterol (mg/dL)	178.3 ± 32.7	180.3 ± 38.6	214.3 ± 45.7	< **0.001**
Triglycerides (mg/dL)	110.8 ± 45.2	108.3 ± 40.1	159.9 ± 50.3	< **0.001**
LDL cholesterol (mg/dL)	103.9 ± 28.5	107.4 ± 33.1	145.7 ± 44.1	< **0.001**
HDL cholesterol (mg/dL)	52.2 ± 14.5	51.3 ± 16.1	36.6 ± 10.4	< **0.001**
MetS				
MetS (number parameter)	0.96 ± 0.8	1.63 ± 0.6	3.83 ± 0.8	**<** **0.001**
Mets presence	3, 5.7%	19, 100.0%	37, 100.0%	χ^2^ = 30.79, ***p*** **<** **0.001**
Ki67 index (%)	2.5 ± 1.8	3.5 ± 4.7	6.1 ± 5.1	**<** **0.001**

GEP-NET: Gastroenteropancreatic tumors; G: grading; BMI: body mass index; WC: waist circumference; SBP: systolic blood pressure; DBP: diastolic blood pressure; HDL: high-density lipoprotein; LDL: low-density lipoprotein; MetS: metabolic syndrome

*A *p*-value in bold type denotes a significant difference (*p* < 0.05)

**Fig. 2:**
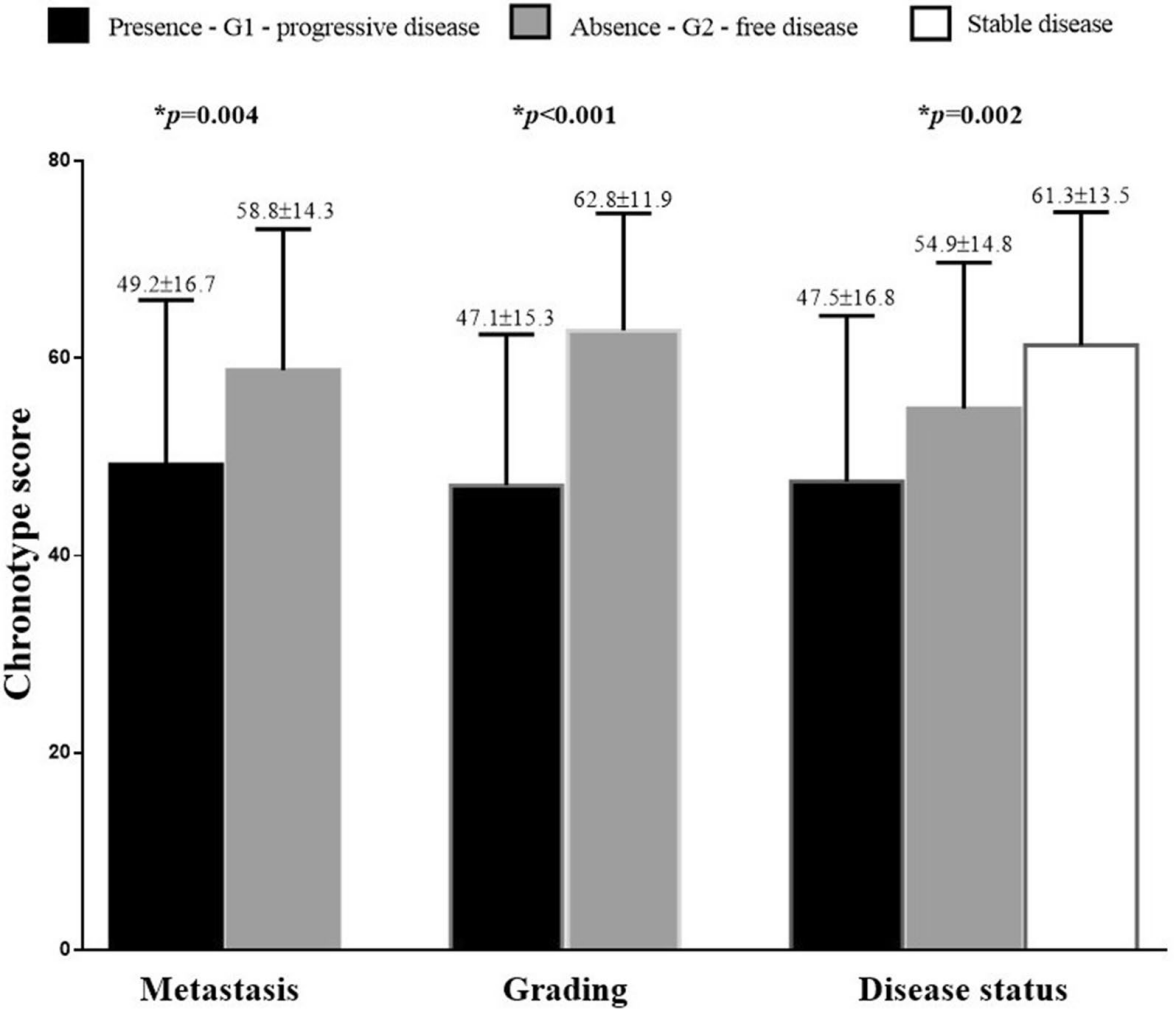
Chronotype score in GEP-NET patients according to the clinical pathological characteristics. Metastatic stage, grading G2 and progressive disease presented the lower chronotype score (*p* = 0.004, *p* < 0.001, and *p* = 0.002; respectively) compared to other categories of the clinical pathological characteristics. Differences in metastasis and grading were analysed by paired Student’s t test, while ANOVA test, with the Bonferroni test as post-hoc test was used to evaluate differences in progressive disease. *A *p* value in bold type denotes a significant difference (*p* < 0.05). G, grading

Interestingly, considering the clinical pathological characteristics, metastatic stage, grading G2 and progressive disease presented the lower chronotype score (*p* = 0.004, *p* < 0.001, and *p* = 0.002; respectively) compared to other categories; Fig. 2.

Correlation analyses were performed to investigate the association of chronotype scores with clinic and metabolic parameters. Chronotype score was negatively associated with all anthropometric measurements, SBP and DBP, metabolic profiles, percentage of MetS, and Ki67 index values (*p* < 0.001 for all) while no correlation was found between chronotype and age (*p* = 0.146) (see Table 3). In addition, these correlations remained significant after adjustment for BMI (*p* = 0.456), Table 3.

**Table 3:** Correlations of chronotype score with study parameters after adjustment for BMI

Parameters	Chronotype score n = 109			
	Simple correlation		After adjustment for BMI	
	r	*p*-value*	r	*p*-value*
Age (years)	− 0.140	0.146	− 0.073	0.456
Anthropometric measurement				
BMI (kg/m^2^)	− 0.469	< **0.001**		
WC (cm)	− 0.511	< **0.001**	− 0.247	**0.010**
Blood pressure				
SBP (mmHg)	− 0.566	< **0.001**	− 0.382	**0.001**
DBP (mmHg)	− 0.489	< **0.001**	− 0.334	**0.002**
Metabolic profile				
Glycemia levels (mg/dL)	− 0.468	< **0.001**	− 0.392	< **0.001**
Total cholesterol (mg/dL)	− 0.400	< **0.001**	− 0.349	**0.001**
Triglycerides (mg/dL)	− 0.485	< **0.001**	− 0.427	< **0.001**
LDL cholesterol (mg/dL)	− 0.493	< **0.001**	− 0.447	< **0.001**
HDL cholesterol (mg/dL)	0.525	< **0.001**	0.495	< **0.001**
MetS				
Mets (number parameter)	− 0.913	< **0.001**	− 0.888	< **0.001**
Ki67 index (%)	− 0.438	< **0.001**	− 0.395	< **0.001**

BMI: Body mass index; WC: waist circumference; SBP:systolic blood pressure; DBP: diastolic blood pressure; HDL: high-density lipoprotein; LDL: low-density lipoprotein; MetS: metabolic syndrome

*A *p*-value in bold type denotes a significant difference (*p* < 0.05)

To assess the correlation of metastases and grading, two bivariate proportional OR models with chronotype score were performed. In the first model, chronotype score was significantly associated with the presence of metastasis (OR = 0.96, *p* = 0.006, 95% CI 0.93 – 0.99, R^2^ = 0.07), and similarly in the second model chronotype score was significantly associated with the highest tumor grade, G2 (OR = 0.93, *p* < 0.001, 95% CI 0.90 – 0.96, R^2^ = 0.23).

A multinomial logistic regression model to assess the association between disease status and chronotype score. Progressive disease was associated with lower chronotype score (χ^2^ = 111.94, *p* < 0.001, R^2^ = 0.64).

**Fig. 3:**
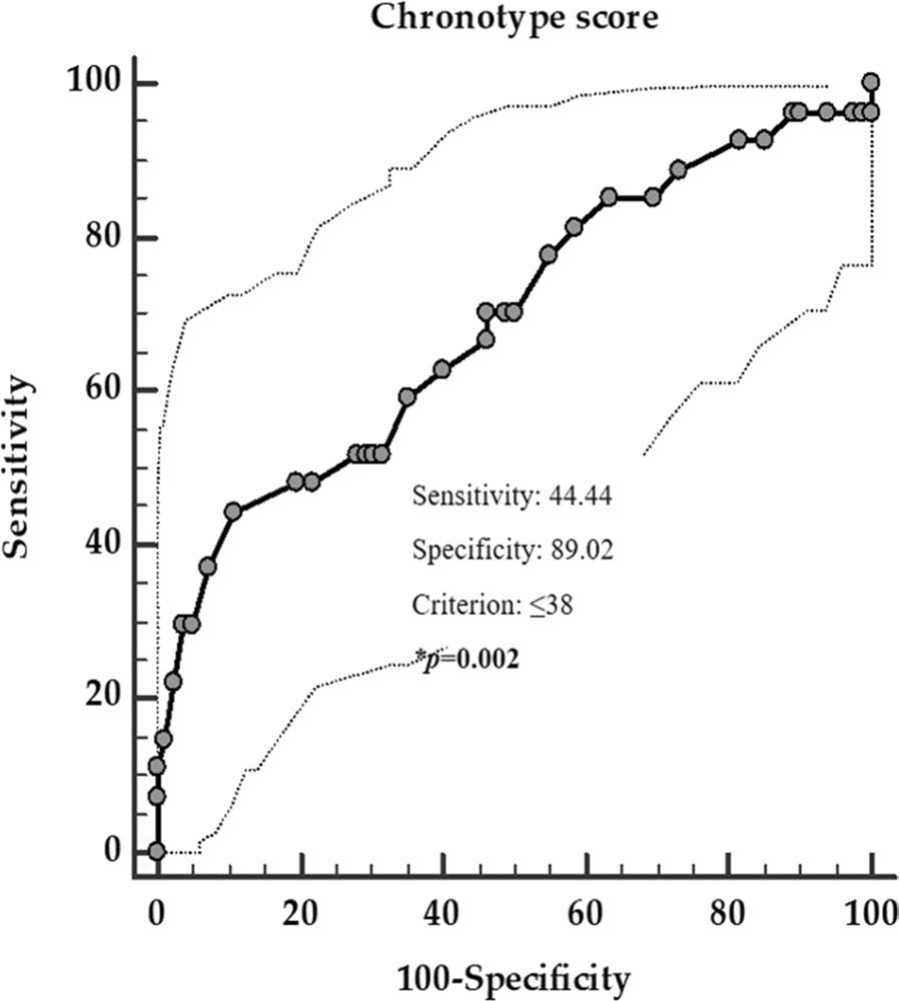
ROC for the value of chronotype score predictive of the presence of metastasis. In the ROC analysis, the threshold value of chronotype score predicting of the presence of metastasis was found at ≤ 38. *A *p* value in bold type denotes a significant difference (*p* < 0.05)

**Fig. 4:**
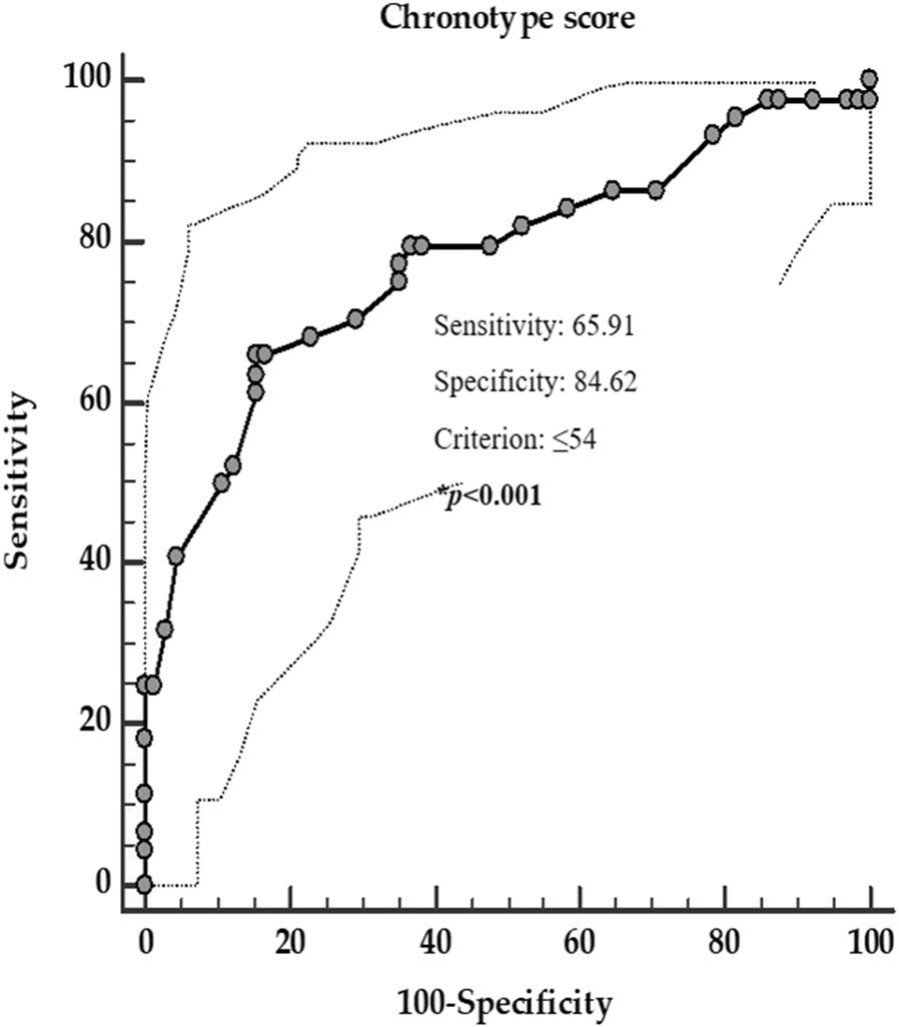
ROC for the value of chronotype score predictive of the highest grading (G2). In the ROC analysis, the threshold value of chronotype score predicting the highest grading (G2) was found at ≤ 54. *A *p* value in bold type denotes a significant difference (*p* < 0.05)

**Fig. 5:**
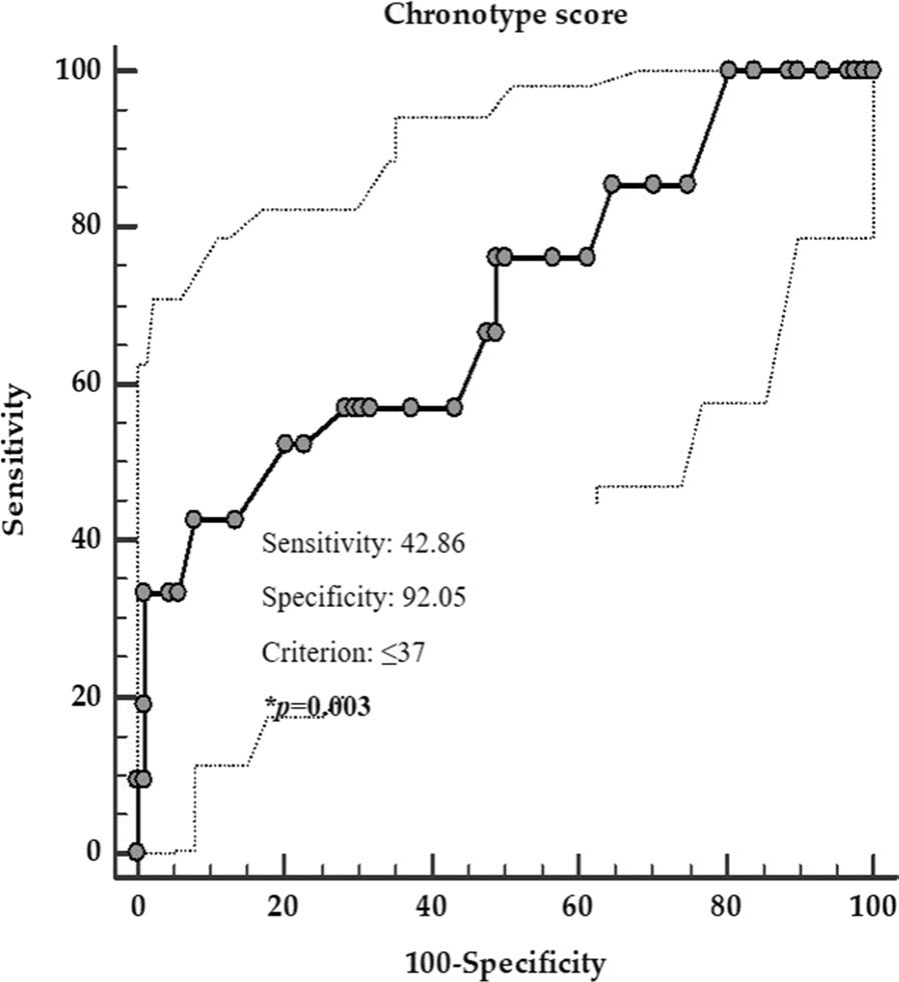
ROC for the value of chronotype score predictive of the highest progressive disease. In the ROC analysis, the threshold value of chronotype score predicting the highest progressive disease was found at ≤ 37. *A *p* value in bold type denotes a significant difference (*p* < 0.05)

Three ROC analysis were performed to determine the cut-off values of the chronotype scores predictive of the presence of metastasis, grading G2, and progressive disease, respectively. A chronotype score ≤ 38 (*p* = 0.002, sensitivity 65.9%, specificity 84.6%, AUC 0.69, standard error 0.06, and 95% CI 0.57 – 0.81, Fig. 3), a chronotype score ≤ 54 (*p* < 0.001, sensitivity 65.9%, specificity 84.6%, AUC 0.77, standard error 0.048, and 95% CI 0.68 – 0.87; Fig. 4), and a chronotype score ≤ 37 (*p* = 0.003, sensitivity 42.8%, specificity 92.1%, AUC 0.70, standard error 0.07, and 95% CI 0.57 – 0.83; Fig. 5), could serve as thresholds for significant increased risk of presence of metastasis, grading G2, and progressive disease, respectively.

## Discussion

In this cross-sectional case–control observational study, we investigate the role of individual differences in the chronotype in the context of GEP-NET. As novel findings, we reported that GEP-NET patients have lower chronotype score and higher percentage of evening chronotype than controls, and presented a worse metabolic profile. Considering the clinical presentation and course, patients with the presence of metastasis, grading G2, and in progressive disease showed a lower chronotype score compared to the absence of metastasis, grading G1, and free/stable disease, respectively. Chronotype score was also inversely associated with all anthropometric measurements, blood pressure, metabolic profile, MetS, and Ki67 index, after adjustment for BMI. Specific cut-off values of the chronotype score have also been provided to detect the increased risk of the presence of metastasis, grading G2, and progressive disease.

Chronotype, the expression of circadian rhythmicity in each individual relative to the external light–dark cycle, is highly variable across the human population, with substantial differences in biological and behavioral parameters. In agreement with our findings, subjects with the evening chronotype have been reported to be more prone to follow an unhealthy lifestyle, performing less regular activities and being more frequently smokers [21]. A higher percentage of MetS have been also detected in evening chronotype. Disruption of the circadian system could result in sleep disturbances and increased appetite [52] that, in turn, increases the risk of developing insulin resistance, the core mechanism of MetS. Furthermore, in animal models, mutations of circadian clock genes can lead to signs of MetS [53]. We also found a worsened cardiometabolic profile in evening subjects. Similarly, results were found in a cross-sectional study carried out in 325 full- and part-time female hospital employees (168 rotating night workers, 157-day workers) [54]. Cardiometabolic risk factors such as higher WC, BMI, glycemia levels, blood pressure, and cardiometabolic risk score were a common finding in evening chronotype [54]. Moreover, subjects with the evening chronotype have been reported to have a lower adherence to the Mediterranean diet [21] as well as it has been found in subjects with aggressive GEP-NET compared to grading G1, localized and free/stable disease status. Low adherence to a healthy nutritional pattern such as Mediterranean diet represents an additional risk for the development of obesity [55]. As well-known obesity is currently considered a known risk factor for cancer [56, 57], and at the same time it is often detected in subjects with the evening chronotype [58, 59]. This association could explain the increased risk of developing obesity–related cancers, such as prostate and breast cancer in the evening chronotype [27]. Although it has been highlighted an increased risk of developing cancer, no studies have been carried out to investigate if the evening chronotype was associated with a worsening severity of cancer. In our study, subjects with GEP-NET with evening chronotype were at more risk of having metastasis, to have the highest grading and progressive disease. The most plausible hypothesis that could explain this association could lie in the fact that obesity, commonly coexisting with the evening chronotype, is often associated with insulin resistance that in turn elicits secondary hyperinsulinemia, growth factor cross-binding, insulin-like growth factor-1 receptor [60, 61]. Moreover, we identified a cut-off of chronotype scores that were associated with metastasis, grading G2 and progressive disease, thus providing a new tool to be used in the endocrinology outpatient clinics as an additional tool to suspect advanced disease in subjects with GEP-NET.

Our study also has several strengths, and the following should be highlighted: (1) As far as we know, the current study is the first to explore the individual differences in chronotype in a group of NET patients compared to a control gender, -age, and BMI-matched and its association with clinical severity in patients with NET. Identifying subjects with GEP-NET, and evening chronotype becomes of paramount importance because they could be at risk of developing the more aggressive disease type, thus they need to be tightly followed up. Furthermore, an intervention trying to align the attitude of the evening chronotype to circadian time might be an attempt to prevent the worsen of the disease; (2) the use of a widely used validated questionnaire for collecting data on the chronotype [21, 62]. Of interest, the MEQ questionnaire allows one to provide feedback to the participants immediately after the interview is completed. To avoid interoperator variability, and to minimize any bias related to the filling of the MEQ questionnaire, it was face-to-face administered and not self-reported only the very same expert nutritionist; (3) we included well-selected NET patients with stringent inclusion/exclusion criteria and the matched controls have been well characterized. In particular, we increased the homogeneity of the cohort of studied patients that improved the power of the study by including NET patients who underwent curative surgery and who were biochemically free of disease for more than 6 months, nonfunctioning treatment-naïve patients or patients who had not partaken of medical treatment. Furthermore, all patients had a diagnosis of well-differentiated G1/G2 GEP-NET; (4) Furthermore, being a monocentric study, participants shared the same geographical area and, likely, similar food availability and dietary consumption patterns, which allowed us to improve the homogeneity of the study sample.

The main strength of our study was the novelty of highlighting the role of the assessment of chronotype in the context of GEP-NET. Identifying subjects with GEP-NET and evening chronotype becomes of paramount importance because they could be at risk of developing the more aggressive disease type, thus they need to be tightly followed up. Furthermore, an intervention trying to align the attitude of the evening chronotype to circadian time might be an attempt to prevent the worsen of the disease. The limit of the study is mostly represented by the cross-sectional experimental design that although highlights the association of evening chronotype with a worsening of oncological features, anthropometric and metabolic characteristics of GEP-NET, it misses to provide any explanation on the causality of this association. Moreover, the suggested cut-off value of chronotype score for identifying the clinical severity of NET should be viewed with caution until data of studies in larger NET patients have become available to perform an appropriate cross-validation. Finally, both genetic and environmental factors, including nutrition and gut microbiota, influence the distribution of chronotypes. Nevertheless, we did not include in this study the gut microbiota or gut-derived metabolites, including trimethylamine N-Oxide [63, 64].

## Conclusions

GEP-NET patients, particularly patients with metastasis, grading G2 and in progressive disease, have an unhealthy metabolic profile and present more commonly an evening chronotype. As a potential translational implication of this study, we propose to also include the assessment of chronotype categories as an adjunctive tool for the prevention of metabolic alterations and tumor aggressiveness of GEP-NET. Clinical intervention studies evaluating the effects of changes of chronotype are mandatory to confirm the relationship between evening chronotype, metabolic alterations, and tumor aggressiveness in GEP-NET.

## Data Availability

All data generated or analyzed during this study are included in this published article.

## Abbreviations

NET: Neuroendocrine tumors
GEP: Gastroenteropancreatic
GEP-NET: Gastroenteropancreatic-neuroendocrine tumors
BMI: Body mass index
MEQ: Morningness-Eveningness Questionnaire
MetS: Metabolic syndrome
WC: Waist circumference
CTS: California Teachers Study
ENETS: European Neuroendocrine Tumor Society
SBP: Systolic blood pressure
DBP: Diastolic blood pressure
HDL: High-density lipoprotein
LDL: Low-density lipoprotein
MEN: Multiple endocrine neoplasia
OR: Odds ratio
IC: Interval confidence
AIC: Akaike information criterion
ROC: Receiver operator characteristic
AUC: Area under the curve
